# Exploring Childhood Lead Exposure through GIS: A Review of the Recent Literature

**DOI:** 10.3390/ijerph110606314

**Published:** 2014-06-18

**Authors:** Cem Akkus, Esra Ozdenerol

**Affiliations:** Department of Earth Sciences, University of Memphis, Memphis, TN 38152, USA; E-Mail: eozdenrl@memphis.edu

**Keywords:** childhood lead poisoning, geographic distribution, screening efforts, risk modeling, GIS

## Abstract

Childhood exposure to lead remains a critical health control problem in the US. Integration of Geographic Information Systems (GIS) into childhood lead exposure studies significantly enhanced identifying lead hazards in the environment and determining at risk children. Research indicates that the toxic threshold for lead exposure was updated three times in the last four decades: 60 to 30 micrograms per deciliter (µg/dL) in 1975, 25 µg/dL in 1985, and 10 µb/dL in 1991. These changes revealed the extent of lead poisoning. By 2012 it was evident that no safe blood lead threshold for the adverse effects of lead on children had been identified and the Center for Disease Control (CDC) currently uses a reference value of 5 µg/dL. Review of the recent literature on GIS-based studies suggests that numerous environmental risk factors might be critical for lead exposure. New GIS-based studies are used in surveillance data management, risk analysis, lead exposure visualization, and community intervention strategies where geographically-targeted, specific intervention measures are taken.

## 1. Introduction

The use of GIS in environmental risk factor studies on childhood lead exposure became a focus of research activity in the late 1990s. This prompted the CDC to develop a guideline for the use of GIS in childhood lead poisoning studies in 2004 [1]. Even though the number of children with elevated blood lead levels (EBLLs) in the U.S. is decreasing, eliminating EBLLs by the year 2020 remains a goal of the U.S. Department of Health and Human Services [2]. The capacity to achieve this goal is conditional on the ability to develop strategies based on geographic areas [3]. Funding is another factor to achieve this goal especially when health departments have limited budgets [4]. Despite significant research on the risk factors affecting childhood lead poisoning (age of housing, urban/rural status, race/ethnicity, socioeconomic status, population density, renter/owner occupancy, housing value, nutritional status), there has not been any review article discussing the GIS-based studies. The purpose of this article is to review previous and current GIS research to understand which methods currently employed have been most effective in the screening strategies and examining spatial epidemiology of childhood lead exposure. Another goal is to identify additional methods in GIS-utilized lead poisoning research that also provide public health practitioners and policy makers the ability to better target lead poisoning preventive interventions. Our review covers the time period from 1991 to 2012 and includes GIS-based studies which were published until the adoption of the toxicity threshold of blood lead levels of 5 microgram per-deciliter (µg/dL) by the CDC [5].

### 1.1. Ecological Studies and GIS Use in Childhood Lead Poisoning

Ecological studies focusing on the distribution of blood lead levels, susceptible populations, and exposure sources have been cited to address childhood lead exposure. Identification of environmental risk factors and understanding of the distribution of the lead in the environment is important for health departments in better targeting at risk populations [6,7,8,9]. Ecological studies modeling risk factors are also valuable because they give insight to public health intervention strategies [10,11,12,13,14,15,16,17,18]. For ecological studies of childhood lead poisoning, one needs to identify sources of lead toxicity and determine environmental risk factors based on the distribution of the toxicants and how children come into contact with them in their daily lives [19,20,21,22,23,24,25,26,27,28]. Children’s bodies absorb lead easily, especially in the brain and central nervous system, making them highly susceptible to the effects of lead poisoning. Sources of environmental lead contamination can be difficult to pinpoint because the pathways to lead absorption are various: (1) deteriorating lead-based paint from walls, windows, and doors; (2) transportation of lead contamination to the house by other means; (3) playing with toys which contain lead; (4) absorption of leaded dust through hand-to-mouth behaivour; and (5) being in polluted environment [29]. The most common pathway could be hand-to-mouth behavior especially among young children, however it is hard to know when and how they interact with lead contamination [30]. Exposure during childhood is thought to be brief, usually until the age of 6 [31]; however, the side effects persist throughout life [32]. Possible sources for lead include: leaded paint, lead contaminated soil, lead in plumbing, automobile exhaust, by-products of both mining and metal working, and various consumer products [18,33,34,35,36]. After the ill effects of lead on people’s health were recognized, lead was first banned in Europe in the early 1900s [37]. Lead use in the US was successively banned in paint (1978) [38], in pipes (1986) [39], and in gasoline (1995) [40]. Environmental lead from these sources has not been completely eliminated. Houses with old pipes and paint, which contaminate the drinking water and surrounding soil, are still a significant source of lead exposure [31,36,41].

Despite being a preventable environmental problem, lead poisoning remains a major health threat and a persistent source of illness in the United States. Its estimated cost is $50.9 billion [42]. Changes in federal laws to limit the use of lead reversed the increasing trend in BLLs of children in the US between 1900 and 1975, but children aged <6 years continued to be exposed to lead [31]. In the US, the threshold of elevated blood lead level (EBLL) for childhood lead poisoning has changed four times over the last four decades. Before 1975, lead concentrations of 60 µg/dL and above were considered elevated. With our increased understanding of lead poisoning, the threshold has lowered to 30 µg/dL in 1975, 25 µg/dL in 1985, 10 µg/dL in 1991, and finally 5 µg/dL in 2012 [43,44,45,46,47]. To date, no safe blood lead thresholds for the adverse effects of lead on children have been identified [31]. GIS use in childhood lead poisoning studies started in the 1990s. In 1992, Wartenberg [48] conducted one of the earliest GIS studies on childhood lead poisoning by focusing on theoretical GIS methodologies rather than data analysis. Public health departments recognized the advantages of GIS in screening, exposure prediction, and mapping cases. Using BLL data for lead poisoning, an increasing number of GIS-based ecological studies have identified risk factors as socioeconomic status (SES) [9,10,11,12,13,14,15,16,17,18,21], year built of housing [7,8,9,10,11,13,15,16,17,18,20,21,23,28], race [11,13,14,16,17,21,23,27,28] and ethnicity [15,16,18].

In lead poisoning studies, GIS was used in various stages from data preparation, to multivariate mapping of BLLs with their risk factors, to spatial and statistical analysis. At the data preparation stage, address geocoding is the most used tool to transfer tabular data sets, such as screened children addresses, into GIS [7,8,9,10,11,12,13,14,15,16,17,18,19,23]. Various GIS functions were used for multivariate mapping of BLLs and risk factors in a limited custom such as linking SES data with screened data records [49,50], map overlays [51,52], distance calculations [53], and hyperlinks to demolishing sites’ photos and city maps for mapping dust-fall lead loadings [54]. More sophisticated spatial methods have also been used such as spatial clustering [15,18,21,24,26], spatial autocorrelation [10,13,15,18,21], spatial regression [14], and risk modeling [10,11,12,13,14,15,16,17,18]. New GIS-based studies are used in surveillance data management, risk analysis, lead exposure visualization, and community intervention strategies where geographically-targeted and specific intervention measures are taken.

### 1.2. Recent Reviews

A review of GIS-utilized studies on childhood lead poisoning has not been conducted. There are some non-GIS based reviews on lead poisoning in relation to cardiovascular diseases [55], resuspension of urban soil [56], multiple risk factors on Hispanic sub-population [57], lead dust from traffic volume [58], leaded gasoline on urbanized areas [59], and exposure to lead in soil dust [60]. We will describe these reviews and summarize what is known and unknown as a source of lead exposure and build on these reviews with our comprehensive review, inclusive of GIS-based studies.

Navas-Acien *et al.* [55] studied lead exposure and cardiovascular disease in 2007. The authors reviewed studies regarding the association between BLLs and blood pressure, lead exposure and clinical cardiovascular disease in the general population, cardiovascular mortality in occupational populations exposed to lead, and lead exposure and intermediate cardiovascular end points. The review found a positive association but not a causal relationship between lead exposure and cardiovascular end points in general and occupational populations. The study also showed suggestive—but not causal—evidence that there is a relationship between lead exposure and heart rate variability. These associations were observed at low level BLLs (well below 5 µg/dL).

Laidlaw *et al.* published two reviews about the relationship between lead in soil and children blood lead levels in 2008 and 2011 [56,60]. In 2008, Laidlaw *et al.* [56] claimed that seasonality could be another source of lead poisoning problems besides paint chips, leaded soil, and pipes. Their review also discussed the study designs of “soil lead” *vs.* “blood lead” studies. They created a statistical model in order to investigate the atmospheric soil seasonality and the prediction model for atmospheric soil in the US. In terms of soil lead topology, they reviewed studies indicating that lead in soil decayed exponentially away from the historical main roads [61,62]. Another study by Mielke *et al.* [63] also suggested that changes in soil lead in the inner city might be better explained with historical lead deposits from traffic than from old housing (leaded paint). In their review in 2011, Laidlaw *et al.* [60] focused on Australian inner cities as they found that there were few studies conducted in the inner cities. The authors suggested that there should be high density soil lead mapping as well as universal screening in older neighborhoods in Australia’s large inner cities.

Brown *et al.* [57] presented literature on sources of lead in Hispanic sub-populations which indicates children with Hispanic origin are at high risk in the population. The authors reviewed the literature for lead poisoning among Hispanic populations based on their location, behavior, and diet. In terms of location, the review suggested that there was a relationship between immigrant populations and lead poisoning. Among studies they reviewed, Cowan *et al.* found that children on the Mexican side of US-Mexico border had higher BLLs compared to the children who lived in the US side of the border. However, poverty could be a confounding factor in the area [57]. Another study [64] in their review showed that 43% of Mexican children had elevated BLLs (≥10 µg/dL) in an area close to the border of El Paso, Texas. Location based studies include migratory farmworkers as well. Another location-dependent behavioral pathway was the consumption of lead glazed pottery bits known as “pica”. This material was being consumed by women during their pregnancy in Mexico due to the belief that this material was helpful for the baby [65,66]. In terms of dietary intake, exposure to lead varies from folk remedies to imported candies. The review also suggested that there was food insecurity among Hispanic subpopulations which may result in iron deficiency which increases lead absorption in the bodies of children.

Mielke *et al.* published two reviews about environmental aspects of lead poisoning in consecutive years 2010 and 2011 [58,59]. In their 2010 review, Mielke *et al.* investigated the effect of traffic on lead poisoning regarding lead emissions and additives used in eight California urbanized areas. The authors used three datasets in order to show the gasoline lead contribution in the environment; annual lead amounts from 1927 to 1984, 1982 lead additive quantities for eight urbanized areas in California, and California fuel consumption data from 1950 to 1982. The review showed that there was a correlation between the lead amount in soil and size of the cities. Community location was also related to the lead amount. Inner cities where high traffic volume occurs had higher amounts of leaded soil compared to the suburbs. The review also showed that the distance decay characteristics of lead in soil were similar throughout the US. There was a strong correlation between children BLLs and lead in soil. Mielke’s review confirmed the relationship between children BLLs and seasonality. Mielke *et al.* found a negative relationship between lead in soil and school erformance of children. In their second review in 2011, they expanded their previous California study to 90 urbanized areas throughout the US. Their findings corroborated the previous findings.

## 2. Methods

A literature search was conducted to identify recent articles discussing childhood lead poisoning and the use of GIS and risk modeling. Several online databases were queried, including JSTOR, CINAHL, Web of Science, ScienceDirect, and PubMed. The following key words were used individually and in combination as inclusion criteria for articles to be considered for this review; children, childhood, pediatric, Pb, lead, poisoning, toxicity, geographic, information, systems, and GIS. Our review covers a 21 year period which includes GIS-based studies published since 10 µg/dL thresholds were first introduced in 1991 until the new threshold of 5 µg/dL in 2012. Initial searches yielded approximately 981 results. The abstracts of these papers were reviewed to confirm applicability. After considering additional exclusion criteria (manuscripts not having BLL data analysis, no GIS use, non-English language, and manuscripts not available as full-text), 23 papers remained.

Reviewed articles were summarized and grouped into five categories: screening methodology design, risk modeling studies, environmental risk factors, spatial analysis of genetic variation, and political ecology. Table 1 presents these studies under each category with GIS methods applied, study region, and common risk factors or major findings (Table 1). The first three categories focus on children’s environment. The fourth category, spatial analysis of genetic variation, focuses on individual’s traits. The last category, political ecology, focuses more on the long term socio-economic process of childhood lead poisoning. Some articles could fall into more than one category. We included articles into the categories where they mostly fit.

## 3. Results and Discussion

All of the reviewed articles obtained their lead toxicity data from health departments. In these studies, blood lead screening data was collected by clinics or health workers without GIS. Data collection methods may vary among states.

### 3.1. Screening Activities

Studies on childhood lead poisoning surveillance that used GIS include Lutz *et al.* in 1998, Reissman *et al.* in 2001, Roberts *et al.* in 2003, and Vaidyanathan *et al.* in 2009 [6,7,8,9]. These studies followed CDC’s guidance on targeted screening [67]. The guidance requires that children at ages of 1 and 2 or ages of 3 and 6 should be tested if they have not been tested before and fall in at least one of the following criteria: residing in a ZIP code in which ≥27% of housing was built before 1950; receiving public assistance from programs such as Medicaid or the Special Supplemental Nutrition Program for Women, Infants and Children (WIC); and whose parents or guardians answer “yes” or “don’t know” to at least one of the questions in basic personal-risk questionnaire.

**Table 1:** Summary of studies with common risk factors and major findings.

GIS Analysis/Citation	Region/Date	Common Risk Factors/Major Findings
*Screening methodology design*		
Overlay analysis, choropleth mapping/[6]	Knoxville, TN/1998	Old housing, and proximity to old roads/The screening data based on the study’s risk criteria thoroughly represents the targeted population.
Address geocoding, overlay analysis, choropleth mapping/[7]	Jefferson, KY/2001	Old housing/Percent children with EBLLs is strongly associated with old housing. The screening data based on the study’s risk criteria does not fully represent the targeted population.
Address geocoding, overlay analysis/[8]	South Carolina/2003	Old housing/EBLLs are strongly associated with old housing. The screening data based on the study’s risk criteria does not fully represent the targeted population.
Address geocoding, overlay analysis, choropleth mapping/[9]	Atlanta, GA/2009	Poverty, old housing/The screeing is strongly correlated with WIC (Special Supplemental Nutrition Program for Women, Infants and Children enrolment) status but not with old housing.
*Risk modeling studies*		
Spatial autocorrelation/[10]	Rhode Island/1997	Old housing, poverty, vacancy, percent screened children, and percent immigrants/Older houses and vacant housing are significantly associated with excessive childhood lead exposure.
Address geocoding, overlay analysis, choropleth mapping/[11]	Durham, NC/2002	Old housing, income, and race/The percentage of African American population, median income, and construction year of housings are significantly associated with childhood lead exposure.
Address geocoding/[12]	Rhode Island/2003	Poverty, education, occupation, wealth/BLLs are strongly associated with poverty but not education level, occupation, and wealth.
Spatial autocorrelation with Simultanious Autoregressive Model (SAR)/[13]	New York/2004	Old housing, race, poverty, population density, education, vacant housing, renting, and seasonality/The age of housing, education level, and percentage of African American population variables are significant predictors of BLLs.
Point in polygon analysis (PIP), address geocoding, and spatial regression/[14]	Syracuse, NY/2007	House value, race/EBLLs are significantly associated with the percentage of African American population and average house value.
Spatial autocorrelation, kriging, Local Moran’s I, and LISA/[15]	Cook, IL/2007	Old housing, income, and minority populations/The authors concluded that the dependent variable is significantly associated with housing age, income, and minority populations.
Address geocoding, risk modeling/[16]	North Carolina/2008	Old housing, race, percent Hispanic, income, poverty, and seasonality/All variables are significantly associated with childhood lead exposure.
Address geocoding, sensitivity analysis/[17]	Michigan/2010	Old housing, race, poverty, race, and education/BLL is associated with children’s immediate environment than a larger area such as a census tract or ZIP code.
Spatial autocorrelation, kriging, Local Moran’s I, and LISA/[18]	Cook, IL/2010	Old housing, income, and minority populations/The authors concluded that the dependent variable is significantly associated with housing age, income, and minority populations.
*Environmental risk factors*		
Address geocoding, choropleth mapping, and overlay analysis/[19]	New Jersey/1992	Proximity to industrial sites emitting lead and hazardous waste sites contaminated with lead, and proximity to roads with high traffic volume.
3-D Surface Modeling/[20]	New Orleans, LA/1997	Old housing, soil lead concentration/Association found between high soil lead areas and neighborhoods where children with EBLLs reside.
Choropleth mapping, overlay analysis, kriging, spatial autocorrelation/[21]	Syracuse, NY/1998	Old housing, race, population density, house value, rent/BLLs are correlated with percentage of children at risk, population density, mean housing value, and percentage of the African American population.
Overlay analysis, choropleth mapping/[22]	Mexico/2002	Proximity to a point-source of lead exposure/There is a significant association between children with EBLLs and their distance to a point-source of lead exposure.
Address geocoding, overlay analysis/[23]	North Carolina/2007	Old housing, race, income, seasonality, water system/There is a correlation between water treatment systems and lead exposure among children.
Overlay analysis and kriging/[24]	New Orleans, LA/2011	Proximity to old and heavily used roads/Lead additives in gasoline had more impact on childhood lead exposure than the dust from leaded paint.
Overlay analysis, buffer analysis, spatial masking/[25]	North Carolina/2011	Proximity to local airports/Significant positive association found between BLLs and the distances to the airport locations. Seasonality, age of housing, median household income and minority neighborhoods are also associated with BLLs.
Overlay analysis and Kriging/[26]	New Orleans, LA/2013	Soil lead concentrations in the old city core/A statistically significant relationship found between BLLs and soil lead level-proximity to old city cores.
*Spatial analysis of genetic variation*		
Choropleth mapping, overlay analysis/[27]	Durham, NC/2005	Race, and genetic vulnerability.
*Political ecology*		
Moran’s I, LISA, and spatial autocorrelation/[28]	North Carolina/2008	Old housing, poverty, tenant farming associated with the production of tobacco, rural African American population distribution.

The questions included in the questionnaire are: “Does your child live in or regularly visit a house that was built before 1950?”; “Does your child live or regularly visit a house built before 1978 with recent or ongoing renovations or remodeling within the last six months?”; and “Does your child have siblings or playmate who has or did have lead poisoning?” Some states had additional questions added to the CDC questionnaire. Lutz *et al.* [6] defined the “at-risk” population based on the questionnaire criteria. The study identified old housing and proximity to old roads as most common risk factors among those screened children. The authors produced three maps using the questionnaire data and census demographics. One of the maps shows the percentage of positive screenings for each census tract and another one displays “at-risk” and not “at-risk” screenings overlaid with the percentage of houses built before 1950. The third map plots EBLL children with the percentage of houses built before 1950. Although the study mapped the exact location of children, the state of Tennessee and some other states recently banned the disclosure of exact locations of the subjects in compliance with the HIPPA guidelines [68,69]. Lutz *et al.* found that the screening data thoroughly represents the targeted population in Knoxville, TN.

Reissman *et al.* [7] used GIS to assist the health department’s decision making on screening activities in Louisville, Kentucky. The study attempts to (1) assess the efficacy of Jefferson County CLPP in surveying “at-risk” children and (2) determine the capability of GIS to find neighborhoods or housing units that pose risks to children. The first part of the study focuses on the childhood lead poisoning problem at the neighborhood level whereas the latter part examines the problem at the household level. Different from the Lutz *et al.* study [6], Reissman *et al.* considered the “at-risk” population as children between 6 and 35 months of age who reside in a home built before 1950 or live in a target zone where more than 27% of houses were built before 1950. The authors compared the percentage of screened children with corresponding target zones by both census tracts and ZIP codes. The study found that the percentage of children with EBLLs is strongly associated with old housing. The study also showed that the significant numbers of children who live in at risk areas were not being tested throughout the county. The second part of the study mapped the children who are younger than 7 years old with confirmed BLL ≥20 µg/dL and the houses where more than one child resides with confirmed BLL ≥20 µg/dL.

Roberts *et al.* [8] conducted a study over targeted lead-screening development using GIS in Charleston County, South Carolina. The authors obtained pediatric blood tests between 1991 and 1998 from Charleston County Lead Poisoning Prevention Program. Construction year of the houses was extracted from The Charleston County Tax Assessor. The authors first geocoded the children BLLs and then the buildings in the tax assessor by using Matchmaker/2000 address geocoding software. After the removal of duplicate building addresses from the tax assessor, the authors merged the two geocoded data sets: children BLLs and tax assessor buildings in Charleston County. Apart from Lutz *et al.* [6] and Reissman *et al.* [7], the authors categorized the housing variable in three categories; pre-1950, 1950–1977, and post-1977 in order to be consistent with the CDC’s recommendations. Lead poisoning prevalence ratios in these time frames were compared. The study also displayed the actual locations of the children who have elevated blood lead levels (10 µg/dL and above). The study found that the children who live in a housing unit built before 1950 are four times more likely to have EBLLs than the children who live in a housing unit built after 1950. The study also found that there is no statistically significant difference between the children who live in a housing unit built between 1950 and 1977, and those who live in a housing unit built after 1977. In terms of screening activities, the study found that some areas with high number of pre-1950 housing were not screened at all.

Vaidyanathan *et al.* [9] developed a methodology to assess neighborhood risk factors for lead poisoning problems in Atlanta, Georgia in 2009. Unlike the studies referred in this section above, this study primarily used the Special Supplemental Nutrition Program for Women, Infants and Children (WIC) enrollments to identify “at-risk” populations. The authors used BLL data of children younger than 3 years of age when their blood was drawn in 2005. Three datasets were used in the study; pediatric blood tests by The Georgia Childhood Lead Poisoning Prevention Program (GACLPPP), the land parcel dataset for 1999 by the Center for GIS at the Georgia Institute of Technology in Atlanta, and census block group-level data from the 2000 US Census dataset. Since the boundary of block groups and neighborhoods did not coincide, the study followed a GIS methodology to transfer the age demographics from block groups to the neighborhood level in order to integrate residential land parcel data and blood lead tests with the demographics at the neighborhood level. The study indicated that only 11.9% of children aged ≤36 months from the city of Atlanta were tested for lead poisoning despite the risk of high lead exposure. The authors created a lead exposure index for the neighborhoods based on housing age and poverty. The poverty measure was calculated based on the number of children who were enrolled to the WIC. Housing age risk levels were composed of pre-1950 and pre-1978. The study reveals that 90% of residential units in Atlanta were built before 1978. These housing units might be an important source of lead exposure since most studies in the literature established a relationship between old housing and lead exposure through leaded paint. The study found that some neighborhoods are having as low as 8% of testing in children for lead poisoning whereas more than 78% of the children lived in housing units built before 1950. Excluding the Lutz *et al.* study, all of the studies in this section demonstrate that corresponding health departments failed to account for “at-risk” populations. The studies also demonstrate that GIS could be an effective tool to target “at-risk” neighborhoods by health departments.

### 3.2. Risk Modeling

This section refers to nine articles on risk model development for childhood lead poisoning [10,11,12,13,14,15,16,17,18]. Sargent *et al.* [10] conducted a census tract analysis over childhood lead exposure in Rhode Island. The study used 17,956 BLL screening records from the children who were aged 0 to 59 months and screened between 1992 and 1993. Because of the small area problem, the authors excluded two of the census tracts where there were very few screening samples. The study used the percentage of children with BLL ≥10 µg/dL as the dependent variable. The population of children for the census tracts was assigned based on census estimates. The study’s final model includes five independent variables which explained 83% of the variance in lead exposure. According to the final model, percentages of screened children, households with public assistance income, houses built before 1950, vacant houses, and recent immigrants are positively associated with the outcome measure. Percentages of houses built before 1950 and vacant houses are significantly associated with the dependent variable. The source of lead exposure in immigrant children was unknown due to the possibility that they could be exposed to lead in their home countries. The study also found that there is no association between the percentage of African American population and high lead exposure in Rhode Island.

Miranda *et al.* [11] used a tax level address geocoding procedure to show high risk areas for North Carolina Childhood Lead Poisoning Prevention Program. The study covers the following North Carolina counties: Buncombe, Durham, Edgecombe, New Hanover, Orange, and Wilson. The authors first geocoded the screened children at the tax parcel unit in order to detect the age of housing from tax assessors datasets. Overall geocoding match rates vary from 47.2% to 72.1% for the six counties in North Carolina. Using this geocoded dataset, the authors employed analysis of variance (ANOVA) and multivariate analysis to find out whether the independent variables (age of the building, median income, and race) are statistically associated with the BLLs. Miranda *et al.* also prioritized the Durham, NC region in four risk areas: (1) predicted parcels which are most likely to contain leaded paint; (2) predicted parcels which are less likely to contain leaded paint; (3) predicted parcels which are lesser likely to contain leaded paint; and (4) predicted parcels which are least likely to contain leaded paint. Unlike the Sargent *et al.* study, Miranda *et al.* found that the dependent variable is correlated with the percentage of the African American population as well as median income and construction year of housings. One major shortcoming of the model is missing data since address geocoding rates may be under 50%. This study was later updated by Kim *et al.* [16] in 2008. The authors investigated how much the additional data from more intensive geocoding processes improved performance of childhood lead exposure risk models in identifying areas of elevated lead exposure. They used a comprehensive three-level stepwise address geocoding process. Similar to the studies by Miranda *et al.* [11], Griffith *et al.* [14] and Kim *et al.* also deployed an address geocoding based on the cadastral parcel reference system. Also similar to the Miranda *et al.* study [11], the geocoding success rate was lower because 31.2% of the addresses were not geocoded. The results in this study support the findings of the Miranda *et al.* [11] study and also find support for the following independent variables: percentage of Hispanic population, percentage of households with public assistance, and seasonality are also strongly associated with BLLs in the studied population.

Kriger *et al.* [12] examined temporal and spatial scale effects and the choice of geographical unit (*i.e.*, census block group, census tract, and ZIP code) to monitor social inequalities in childhood lead poisoning. The authors used blood lead level screenings of children who live in Rhode Island. The screening period was between 1994 and 1996. Different from Miranda *et al.* [11], Kriger *et al.* used a street reference system (known as Topologically Integrated Geographic Encoding and Referencing (TIGER) dataset) for their address geocoding process. Street reference systems generally produce higher geocoding success rates compared to cadastral parcel reference systems. For instance, the Kriger *et al.* study produced more than 90% of geocoding success in all geographic units, census block groups, census tracts, and ZIP codes. However, one potential weakness of the method is that street geocoding results may be distant from the actual location of houses since the method uses a linear interpolation on street segments in the reference file. The authors found that the choice of measure and the level of geography matter. Census tract and census block group socioeconomic measures detected stronger socioeconomic gradients than the zip code units. The results indicate that BLLs are strongly associated with poverty but not education level, occupation, and wealth. A similar sensitivity analysis was conducted by Kaplowitz *et al.* [17] in 2010. Kaplowitz *et al.* assessed predictive validity of different geographic units for their risk assessment. According to their study, census block groups explain more variance in BLL than high and low risk ZIP codes. Their study confirmed that children’s BLL is more closely associated with characteristics of their immediate environment than with characteristics of a larger area such as a census tract or ZIP code.

Haley and Talbot [13] presented a spatial analysis of BLLs in New York for the children born between 1994 and 1997. The study used the highest test result when there are multiple screens for a child. The authors obtained the birth records from the NYSDOH Bureau of Vital Statistics for the years between 1994 and 1997. Since the BLL records contain ZIP codes for the children, the authors used ZIP codes as the geographic units for spatial analysis. Apart from the other studies mentioned in this section, address geocoding was employed at ZIP level. Based on previous studies in the literature, Haley and Talbot selected the following socioeconomic variables: the percentage of houses built before 1940 and 1950, the percentage of adults ≥25 years of age who did not receive a high school diploma, the percentage of children living below the poverty level, the percentage of vacant housing units, the percentage of the population that rents a home, the percentage of the population screened in summer (July–September), population density, and the percentage of African-American births. The authors also used GIS to distribute the socio-economic data proportionally to the ZIP codes and to find the centroid locations of census blocks. In order to deal with missing data in the lead database, the authors used the mother’s race from birth certificates and estimated the proportion of African American children for each ZIP code area. Unlike Sargent *et al.* [10], this study used a different methodology to deal with the small area problem. Using GIS, the authors merged the ZIP code areas when they have less than 100 screened children. Percentage of children with EBLLs in each ZIP code was defined as the dependent variable in the statistical analysis. The authors ran a multiple linear regression analysis to identify the relationship between the BLLs and the explanatory variables. They also analyzed the residuals’ spatial autocorrelation in the model using SpaceStat software and developed a simultaneous autoregressive model (SAR). Their regression analysis indicates that the age of housing, education level, and percentage of African American population variables are significant predictors of BLLs.

Griffith *et al.* [14] conducted an address geocoding study in 2007. The authors used BLLs data of children in Syracuse, NY between 1992 and 1996. The study compares two different address geocoding methods to find the impact of positional accuracy on spatial regression analysis of children’s BLLs. These geocoding methods are based on street or polygon reference systems. The Haley and Talbot referred above used ZIP code boundaries as the polygon reference system. Griffith *et al.*, on the other hand, used cadastral parcels as the reference files. Geocoding success rate is generally much higher in geocoding process with street reference files than ones with cadastral parcel reference files. However, cadastral parcel reference files provide more precise geocoding results and the construction year of housing units. The authors compared cadastral and TIGER based geocoded addresses in three sections including, census tract, census block group, and census blocks of 1990 and 2000 census demographics. The study shows that there is a noticeable but not considerably high positional error difference in their spatial statistical analyses using the two methods. The regression analysis in the study was employed in two different BLL thresholds, 5 and 10 µg/dL. Regardless of the threshold level, the results indicate that EBLLs are significantly associated with the percentage of the African American population and average house value in the census block and census block group analyses.

Using descriptive discriminant and odds ratio analyses, Oyana *et al.* [15,18] created a profile of high-risk areas based on housing age, the socioeconomic status, and ethnicity of the population in Chicago. The purpose of the study is to identify the health disparity among children who have different racial make-up. The study also assesses the spatiotemporal dynamics of the disease and identifies the socio-economic and racial composition of high-risk communities in Chicago. In addition, two different types of blood test methods (capillary and venous) were compared to one another for the BLL over 10 µg/dL. Oyana *et al.* uses a GIS scripting tool to deduplicate pediatric blood data. This study also differs from others by producing a kriging map for the area. The kriging map of Chicago shows that Westside area has the highest risk of EBLLs in the city. The authors also used TerraSeer’s Space-Time Intelligence Systems (STIS) to explore the krigged prevalence rates in order to analyze spatial patterns [70]. Moran’s I [71] and LISA statistics [72] were used with spatial autocorrelation to show the spatial patterns and health disparities in childhood lead toxicity in Chicago. The variations in raw prevalence rates for BLLs were high. However, kriging reduced the variations dramatically. The authors concluded that the dependent variable is significantly associated with housing age, income, and minority populations.

### 3.3. Environmental Risk Factors

This section discusses eight studies that address environmental risk factors [19,20,21,22,23,24,25,26]. Guthe *et al.* [19] conducted one of the earliest GIS studies on childhood lead poisoning in 1992. The authors studied New Jersey municipalities of Newark, East Orange, and Irvington. The study mapped blood screening records overlaid with census tracts in the municipalities. Children blood samples were from the years 1983 to 1990. Unlike all the relevant studies reviewed in this article, the study used a 15 µg/dL threshold level, which was the BLL threshold level at the time. This study used street level address geocoding. Guthe *et al.* used command line address matching software, which is one of the oldest address geocoding engines. In terms of environmental factors, Mielke *et al.* [20] studied the associations between childhood BLLs and soil lead in Louisiana. The study used three data sets: soil lead data, age of housing data, and children blood lead data for urban New Orleans and rural Lafourche Parish in Louisiana. The study focused on soil contamination and leaded paint sources of lead toxicity problems. The percentage of housing built before 1940 was considered an indicator of leaded paint. Using x and y coordinates of census tract centroids, the authors plotted the three data sets within a three dimensional spatial model. The study showed that there is a relationship between low BLLs and new housing neighborhoods, and old housing neighborhoods were split evenly between old and new housing. There is also an association between high soil lead areas and neighborhoods where children with EBLLs reside. The study suggests that inner-city children should be the focus area to eliminate lead toxicity problems in the population.

Griffith *et al.* [21] employed several GIS tools that include geocoding, buffer analysis, and interpolation techniques such as kriging to depict the lead poisoning problem in Syracuse, NY. This study shows the geographic distribution of lead toxicity in Syracuse, NY in three aggregated levels: census block, census block group, and census tract. Linear regression with spatial autocorrelation is used as a statistical method for the three aggregated levels. The study shows that there is a major difference between urban and rural exposure, which is consistent with the results from Laidlaw *et al.*, and Mielke *et al.* [56,58,59]. It however finds no statistically significant relationship between historically heavily traveled streets and lead exposure. Lead poisoning is detectable regardless of the level of geographic resolution. Griffith *et al.* also showed that BLLs are correlated with percentage of children at risk, population density, mean housing value, and percentage of the African American population.

Gonzalez *et al.* [22] investigated the possible impact of point sources of lead exposure relative to other types of lead exposure sources. The study was conducted in Tijuana, Mexico with, Hispanic children aged between 1.5 and 6.9 years. In order to deal with the confounding variable of cultural habits, the study used BLLs where the subjects reported that they did not use lead-glazed ceramics for cooking or food storage purposes. The study was composed of 76 samples from 14 sites. Gonzalez *et al.* mapped the distribution of these 76 point sources as well as five point sources containing 19 soil samples with the values ranging from 100 to 7870 µg/g soil lead. They compared the children BLLs with Bocco and Sachez [73] study’s prediction model which was based on fixed industrial lead point sources. Similar to the Bocco and Sachez study, the authors assigned Tijuana census tracts the labels of “high”, “medium”, “low”, and “N/A” risk levels based on proximity to the lead point sources. The authors also mapped these risk levels of census tracts and children cases with elevated blood lead levels (≥10 µg/dL) where the subjects reported non-use of lead-glazed ceramics.

In 2007, Miranda *et al.* [23] explored the potential effect of the use of chloramines in water treatment systems over childhood lead exposure in Wayne County, North Carolina. The authors examined the relationship between these potential effects and the age of housing in order to help guide policy practices in North Carolina. The authors used the datasets of children BLLs, tax parcels, census data, and water treatment system boundaries. Children BLLs were geocoded based on tax parcels with a 72.4% geocoding success rate from the surveillance data between 1999 and 2003. The study used multivariate regression to analyze the data and concluded that the use of chloramines in the water treatment systems might inadvertently increase lead exposure among children.

Another environmental study by Miranda *et al.* [25] conducted in 2011 to investigate the relationship between avgas lead exposure and children BLLs. The authors selected 66 airports in 6 counties of North Carolina based on the availability of tax assessor data, the volume of air traffic, and the number of screened children for lead toxicity. The study used the airports’ estimated annual lead emissions which were obtained from the U.S. EPA Office of Transportation and Air Quality. The children BLL data composed of the blood tests conducted between 1995 and 2003 for the children between the ages of 9 months and 7 years. The authors determined the airport boundaries using tax parcel data. The authors created buffer zones surrounding each airport selected in the study. The buffers were created based on the distances of 500 m, 1000 m, 1500 m, and 2000 m from the polygon edges of the airports. Unlike most of the studies discussed in this review article, Miranda *et al.* used GIS to show children locations in a jittered representation even though they run the statistical model based on actual point locations. Using the geocoded locations, Miranda *et al.* was able to join children locations and buffer zones, which were created from the airport boundaries. The authors assigned dummy variables to children locations based on the boundaries mentioned above and seasons for the screening time. The model includes the age of housing, screening season, and demographic variables. The authors also used inverse population weights to eliminate the possible bias caused by high numbers of screening cases on parcels. The study found a significant positive association between logged BLLs and the distances to the airport locations. It further shows that seasonality is an important factor in estimating BLLs. In fall, spring, and summer seasons, children were found having higher BLLs on average compared to winter season screenings. Age of housing was negatively associated with BLLs while the median household income and minority neighborhoods had positive associations with BLLs.

Mielke *et al.* [24] conducted a comparative analysis of lead poisoning problems by assessing the pre-Katrina blood and soil lead concentrations around public and private properties in New Orleans. Soil lead data was composed of 587 soil samples (224 samples from public properties, and 363 samples from residential private properties) and 55,551 BLL screening records for the years between 2000 and 2005. The study shows significant differences among the blood lead prevalence between the inner city (CJ Peete) and outlying areas (Florida) of New Orleans. The study also found no statistically significant other differences between inner and outer cities. The authors found that, among the screens in public properties, differences between inner and outer cities in lead toxicity prevalence are a better proxy than age of construction. The study noted that lead additives in gasoline had more impact on childhood lead exposure than the dust from leaded paint. In terms of lead dust from vehicles, the largest amount of lead was deposited on soil in the inner-cities whereas outer-cities were not experiencing a large amount of lead deposit from the exhaust due to a lighter traffic volume. Consequently, the study indicated that lead toxicity originated from soil contamination could help explain lead toxicity in children.

In 2013, Mielke *et al.* [26] analyzed the association between children blood lead levels and soil lead concentrations in relation to before and after hurricane Katrina in New Orleans. In the study, pre-Katrina was from 2000 to 2005 and post-Katrina was from 2006 to 2008. Children’s blood samples (55,551 records in pre-Katrina and 7384 records in post-Katrina period) were geocoded at the 1990 census tract level. Soil lead data was composed of 5467 soil samples. Soil samples were categorized by their one meter proximity to “busy streets”, “residential streets”, “house sides”, and “open spaces”. Census tract medians of soil lead concentration data were used to produce kriging maps of soil lead concentration for both pre- and post-Katrina periods. Census tracts were also categorized as low and high in lead concentration groups based on 100 mg/kg threshold (≥100 mg/kg and <100 mg/kg). Non-parametric statistics were used because of positive skewness in the soil lead data. Multi-purpose permutation procedure showed that there was a significant difference between low and high lead tracts. This confirms the significance of 100 mg/kg as a threshold for lead concentration in soil for New Orleans. Census tract soil lead concentration medians showed that busy streets had the highest median by location. This could be related to historical lead deposits from car exhausts. Kriging maps showed that there was no major change in the lead concentration level in soil for pre- and post-Katrina periods. Unlike Griffith *et al.* [21], this study suggests that there is a statistically significant relationship between BLLs and soil lead level-proximity to old city cores.

### 3.4. Genetic Variation

One of the reviewed studies focused on the genetic variation of childhood lead poisoning problems [27]. Since other studies found a significant relationship between childhood lead poisoning and African American populations, the authors focused on genetic variation of the problem. The study used previously developed data of children BLLs by Miranda *et al.* [11], which geocoded children cases at the tax parcel level in order to get the construction year of house units from tax assessor data. The study also considers the occupancy status, which was also gathered from tax parcels. The authors note that the spatial autocorrelation problems were minimized by assigning individual year of construction from tax parcels. The ANOVA comparison of models with and without spatial autocorrelation also corroborated the non-existence of spatial autocorrelation. Since some of the information pertaining to construction years is missing in the tax parcel dataset, some cases lacked this information. In those cases, the study assigned the construction year from the nearby parcels. Some studies in the literature indicate that the relationship between high BLLs and African American populations might be because of low calcium intake in the population. According to this study, however, the relationship between high BLLs and African American populations might be more related to genetic polymorphisms.

### 3.5. Political Ecology

Hanchette’s study focused on the political ecology aspect of childhood lead toxicity. The author used Moran’s I [71] and LISA statistics [72] to investigate the spatial distribution of lead poisoning prevalence at the county level in North Carolina. They use 10-year children BLL data from 1995 to 2004. In the study, the data findings show that there is a significant cluster of high BLL rates in eastern North Carolina. The author indicated that these clusters of high rates show persistent health disparities in the region. Hanchette claims that the health disparities in eastern North Carolina results from large scale socio-economic and cultural processes rather than neighborhood characteristics such as poverty and old housing. The study found that the Appalachia (western North Carolina) region displayed low rates of lead poisoning even though the region had high poverty rates. Another major finding is that high rates of lead poisoning clusters correspond with African American populations only in eastern North Carolina. Unlike this region, southern North Carolina does not have high rates of lead poisoning despite high concentration of African American populations. The author suggests that the convergence of poverty, older housing, and the large rural African American population can be explained by the long history of tenant farming. According to Hanchette, this transition from an agricultural state to a mixed economy led to changes in socio-economic characteristics of the eastern region of North Carolina.

## 4. Conclusions

This article reviewed twenty-three GIS-based studies examining spatial modeling of childhood lead poisoning and risk factors that were published after 1991, the year the CDC’s threshold updated to10 µg/dL. GIS use in lead studies revealed greater detail about the magnitude of lead poisoning within populations. Reviewed articles indicate that surveillance and screening practices have extended considerable amount of importance in targeting “at-risk” populations. However, the literature shows that some health departments failed to account for “at-risk” populations [7,8,9]. This issue can be resolved through the implementation of GIS in health departments.

Risk factors for childhood lead poisoning (age of housing, urban/rural status, race/ethnicity, socioeconomic status, population density, renter/owner occupancy, housing value, and nutritional status) have been thoroughly parsed out in childhood lead poisoning research. Unfortunately, address geocoding methods, the parameters used, and the uncertainties they presented were not included in a similar level of detail in the research. Most of the reviewed studies did not provide the input parameters such as the reference system and the match rate. Since these parameters have a direct impact on results of the spatial analyses, this makes it difficult to conduct legitimate comparisons among the various articles.

Even though to date no safe blood lead thresholds for the adverse effects of lead on children have been identified [31], data related to children with very low BLLs has consistently been overlooked. Address information of children with BLLs ranging from 0–3 µg/dL may not be reported since screening efforts have primarily focused on children with high BLLs [74]. This non-random missing data can cause misinterpretation of the spatial distribution of lead poisoning. In order to improve the quality of geocoding, the addresses need to be confirmed in the data collection phase of a GIS environment. Such GIS-integrated screening could eliminate spatial bias due to disparities in reporting. Future studies are needed to fill this gap and attempt to improve the use of address geocoding in BLL data collection.

Future lead poisoning studies should also be concerned with data aggregation and the choice of geographical analysis. Data aggregation is done for two reasons: to link socio-economic and environmental measures to lead data and to ensure data confidentiality. In the former case, geocoded addresses may fall far away from their actual locations resulting in boundary problems during data aggregation to census block groups, census tracts, or ZIP code areas. Very few studies examined these aggregation problems and spatial scale effects to monitor risk factors [14]. Studies show that finer geographic units such as census block group levels explain lead poisoning problems better, and hence some high levels of data aggregation (such as ZIP codes or census tracts) may not explain the distribution in the population [12,17]. Moreover, longitudinal lead studies are subject to possible errors as a result of change in census boundaries over time. In the latter case, very few studies examined the use of GIS and developed techniques to preserve confidentiality during the process of dissemination of screened children data and the resultant high risk areas [25].

Environmental studies on lead paint usage before 1978 have shown a link between house age and elevated BLLs. Soil studies can also reveal sources of lead toxicity. Several studies have shown that the distribution of lead toxicity among young children can be explained by proximity to high volume traffic areas. The relationship of vehicular lead deposits and children with elevated BLLs is contentious. Griffith *et al.* [21] found no relationship between childhood lead toxicity and their proximity to heavily traveled roads. Contrary to Griffith’s findings, Mielke *et al.* [20,24,26] found that childhood lead poisoning was related to residing in inner-city areas where the traffic flow was historically larger. Miranda *et al.* [25] also found a correlation between the proximity of airports and BLLs among children. None of the reviewed studies accounted for housing abatement efforts in their models. Future studies focusing on environmental lead sources need to factor in abatement efforts that may have taken place. By factoring in housing abatement efforts we can eliminate erroneous data and misinterpretations.

The environmental studies in this review also indicate a correlation between BLLs and African American populations. However, very few studies investigated the individual characteristics of children [27]. The history of socioeconomic and cultural processes could also be important factors to identify risk areas [28]. More GIS-based studies need to be conducted to investigate these factors. All of the articles reviewed in this paper show the development of an increasing awareness of the intricacies of lead poisoning and its effects on children and their neighborhoods.
